# *Croton gratissimus* Burch Herbal Tea Exhibits Anti-Hyperglycemic and Anti-Lipidemic Properties via Inhibition of Glycation and Digestive Enzyme Activities

**DOI:** 10.3390/plants13141952

**Published:** 2024-07-17

**Authors:** Veronica F. Salau, Kolawole A. Olofinsan, Abhay P. Mishra, Olufemi A. Odewole, Corinne R. Ngnameko, Motlalepula G. Matsabisa

**Affiliations:** 1 Department of Pharmacology, Faculty of Health Sciences, University of the Free State, Bloemfontein 9300, South Africa; veronica.salau@yahoo.com (V.F.S.); kollyck@gmail.com (K.A.O.); mishra.ap@ufs.ac.za (A.P.M.); ngnameko.cr@ufs.ac.za (C.R.N.); 2 Department of Pure and Industrial Chemistry, Faculty of Physical Sciences, University of Nigeria, Nsukka 410001, Enugu State, Nigeria; abiola.odewole@unn.edu.ng

**Keywords:** glycation, hyperglycemia, obesity, digestive enzymes, herbal tea

## Abstract

Over the years, the world has continued to be plagued by type 2 diabetes (T2D). As a lifestyle disease, obese individuals are at higher risk of developing the disease. Medicinal plants have increasingly been utilized as remedial agents for managing metabolic syndrome. The aim of the present study was to investigate the in vitro anti-hyperglycemic and anti-lipidemic potential of *Croton gratissimus* herbal tea infusion. The inhibitory activities of *C. gratissimus* on carbohydrate (α-glucosidase and α-amylase) and lipid (pancreatic lipase) hydrolyzing enzymes were determined, and the mode of inhibition of the carbohydrate digestive enzymes was analyzed and calculated via Lineweaver–Burk plots and Michaelis Menten’s equation. Its effect on Advanced Glycation End Product (AGE) formation, glucose adsorption, and yeast glucose utilization were also determined. High-performance liquid chromatography (HPLC) was used to quantify the possible phenolic compounds present in the herbal tea infusion, and the compounds were docked with the digestive enzymes. *C. gratissimus* significantly (*p* < 0.05) inhibited α-glucosidase (IC_50_ = 60.56 ± 2.78 μg/mL), α-amylase (IC_50_ = 35.67 ± 0.07 μg/mL), as well as pancreatic lipase (IC_50_ = 50.27 ± 1.51 μg/mL) in a dose-dependent (15–240 µg/mL) trend. The infusion also inhibited the non-enzymatic glycation process, adsorbed glucose effectively, and enhanced glucose uptake in yeast cell solutions at increasing concentrations. Molecular docking analysis showed strong binding affinity between HPLC-quantified compounds (quercetin, caffeic acid, gallic acid, and catechin) of *C. gratissimus* herbal tea and the studied digestive enzymes. Moreover, the herbal tea product did not present cytotoxicity on 3T3-L1 cell lines. Results from this study suggest that *C. gratissimus* herbal tea could improve glucose homeostasis and support its local usage as a potential anti-hyperglycemic and anti-obesogenic agent. Further in vivo and molecular studies are required to bolster the results from this study.

## 1. Introduction

The world has hitherto been plagued by type 2 diabetes (T2D), a metabolic disorder typically defined by chronic hyperglycemia and glucose intolerance, with concomitant altered metabolism of carbohydrates, fats, and proteins, resulting from inadequate insulin secretion and impaired efficacy of insulin to stimulate glucose uptake [1]. In 2021, over 537 million adults were reported to be living with diabetes, with 6.7 million associated deaths. Diabetes cases are estimated to increase to 643 and 783 million in 2030 and 2045, respectively, if not curbed [2]. As a lifestyle disease, obese individuals are at higher risk of developing the disease, keeping diabetes incidences on the rise [3].

Excess ingestion of carbohydrates and physical inactivity have been linked with T2D and obesity, which are associated with increased hepatic glucose production, free fatty acid release via lipolysis, and insulin resistance. Insulin resistance incapacitates peripheral tissues from utilizing glucose, thus leading to incessant hyperglycemia [4]. Chronic hyperglycemia further results in non-specific protein glycation. Glycation has been implicated in a cascade of diabetic micro- and macrovascular complications, such as neuropathy, retinopathy, and cardiovascular complications [5]. The effective regulation of blood glucose homeostasis is therefore vital for the prevention of diabetes and the development of its complications.

As a multifaceted disease, diverse therapeutic approaches, including dietary modifications, inhibition of nutrient digestive enzymes (such as α-glucosidase and lipase), and inhibition of the glycation process, have been employed for the management of diabetes. Acarbose, orlistat, and aminoguanidine are common commercial drugs that inhibit α-glucosidase, lipase, and glycation, respectively. However, due to the medical side effects reported on these drugs, coupled with their unaffordability, especially in developing African countries, the use of natural products as safer and more cost-effective alternatives has massively increased [5,6].

Medicinal plants, with their reservoir of natural phytoconstituents, have notably gained prominence for their numerous biological activities and therapeutic health benefits. Belonging to this array of medicinal plants is *Croton gratissimus*. *C. gratissimus* Burch var. *gratissimus* is a tropical African tree or shrub that belongs to the botanical family of Euphorbiaceae. It is commonly known as lavender croton or lavender fever berry. *C. gratissimus* is widely distributed in different provinces of South Africa. The Zulus call it Ihubeshane-elikhulu, Batswana calls it Moologa, and Afrikaans calls it Rekstokbos [7,8]. Its herbal mixture is traditionally used for treating different ailments in communities in South Africa, including colds, rheumatism, candiditis, nasal congestion, and bleeding gums [7,9]. The milk infusion of the bark is popularly used as a purgative by the Zulu community [10]. Urinary tract infections, malaria, diabetes, arthritis, fever, and impotence are other ailments that have been traditionally managed with *C. gratissimus.*

Pharmacologically, earlier studies have reported the antibacterial and antiviral [11], neuroprotective [12], and hepatoprotective [13] activities of *C. gratissimus.* Additionally, the lipid-lowering effect of the ethanolic extract of *C. gratissimus* in streptozotocin-induced diabetic rats has been reported [14]. Likewise, the antidiabetic activity of its ethanolic extract in alloxan-induced diabetic rats was reported by Okonkon, Bassey, and Obot [8]. Studies have shown that, over the years, the medicinal importance of Croton species has been attributed to its secondary metabolites [15]. The presence of phytochemicals including polyphenols, flavonoids, tannins, saponins, glycosides, and carotenoids has been reported in the *C. gratissimus plant* [15]. LC-MS analysis from our previous study [13] also revealed, among others, the presence of alkaloids, flavonols, and glucuronide. Constant consumption of plant polyphenols in the diet has been reported to delay the onset of diabetes [16].

Though the antidiabetic activity of various organic solvent extracts of *C. gratissimus* has been studied in vivo, the present study is aimed at investigating the in vitro anti-hyperglycemic and anti-lipidemic activity of a hot water infusion of *C. gratissimus* herbal tea and the polyphenolic compounds that may be responsible for its activities, in line with its traditional use as a therapeutic herbal tea or decoction. This is to gain deeper knowledge on its probable in vitro antidiabetic mechanisms and support the popular notion that “Tea is an Elixir of Life” by consuming healthy herbal teas.

## 2. Results

As shown in Table 1, HPLC analysis of *C. gratissimus* herbal tea leaf extract revealed the presence of gallic acid, caffeic acid, catechin, and quercetin, with retention times of 4.691, 3.403, 4.567, and 3.522 min, respectively, corresponding to peak areas of 13.5%, 5.2%, 16.4%, 15.6%, and 15.9%. According to what we observed, the gradient method separated polyphenolic compounds from samples fairly well as compared to the retention times of standards, viz. 4.753 (gallic acid), 3.394 (caffeic acid), 4.548 (catechin), and 3.594 (quercetin). It was also interesting to find that a 35 min short run time at 254 nm was sufficient to separate gallic acid and catechin, while a 70 min run time well separated caffeic acid and quercetin at 254 and 300 nm, respectively.

**Figure 1:**
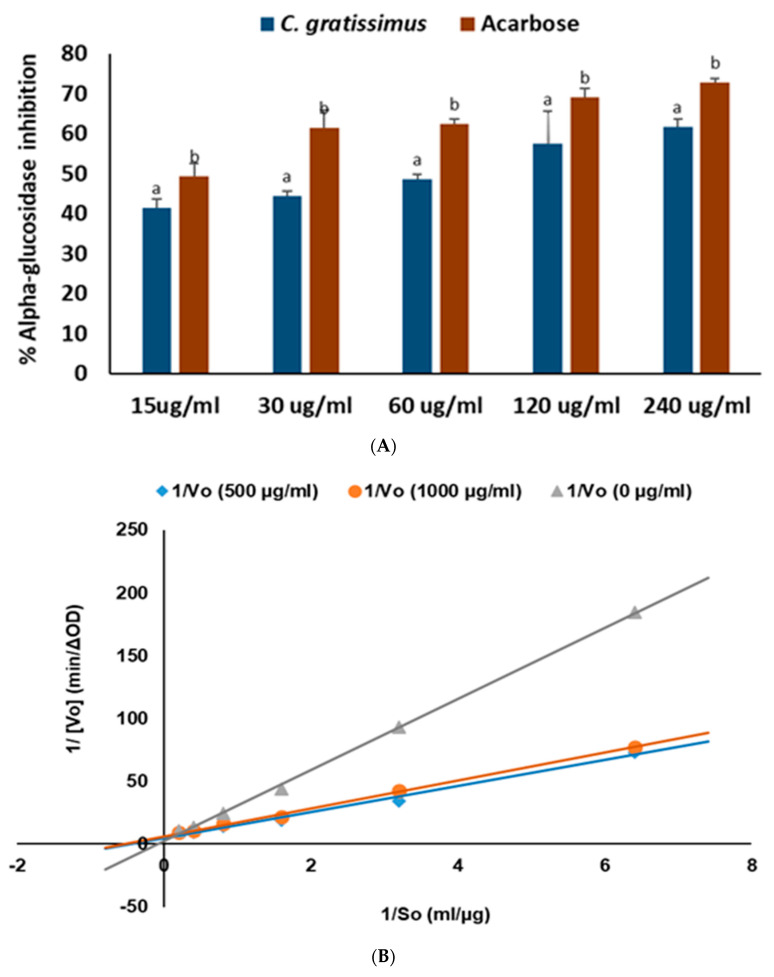
(**A**) α-Glucosidase inhibitory activities of *C. gratissimus* tea and (**B**) Lineweaver–Burk plot for *C. gratissimus* tea mode of *α*-glucosidase inhibition. Data = mean ± SD; n = 3. ^ab^ Values with different letters above the bars for a given concentration are significantly (*p* < 0.05) different from each other.

**Figure 2:**
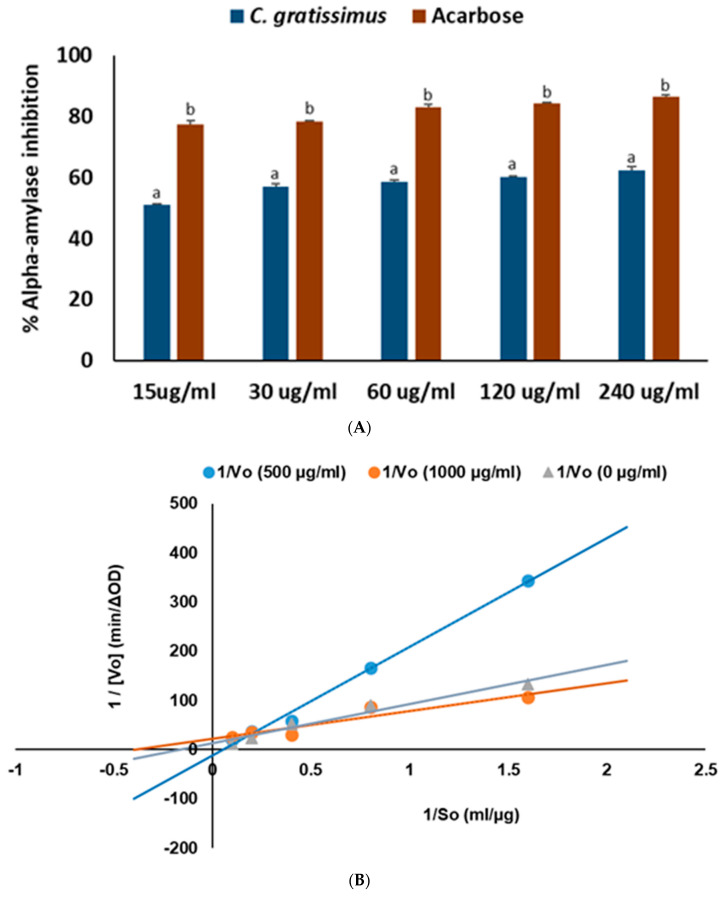
(**A**) α-Amylase inhibitory activities of *C. gratissimus* tea and (**B**) Lineweaver–Burk plot for *C. gratissimus* tea mode of α-amylase inhibition. Data = mean ± SD; n = 3. ^ab^ Values with different letters above the bars for a given concentration are significantly (*p* < 0.05) different from each other.

As shown in Figure 1A, *C. gratissimus* tea significantly (*p* < 0.05) inhibited α-glucosidase activity at all concentrations, with an IC_50_ value of 60.56 ± 2.78 µg/mL (Table 2). The tea also inhibited α-amylase activity (Figure 2A) at an IC_50_ value of 35.67 ± 0.07 µg/mL, although not as obviously dose-dependent as found in the α-glucosidase assay. However, acarbose had a better inhibitory performance on these enzymes by producing, respectively, 25.21 ± 1.19 µg/mL and 9.53 ± 0.18 µg/mL lower IC_50_ values, as revealed in Table 2. The result of the Lineweaver–Burk plot of the digestive enzyme inhibitions (Figure 1B and Figure 2B) was used to calculate the Vmax and Km parameters presented in Table 3. These respective parameter estimates for the inhibition of the infusion on the α-amylase enzyme changed from 0.087 ΔOD/Min and 19.075 µg/mL at 100 µg/mL to 0.046 ΔOD/Min and 2.614 µg/mL at 1000 µg/mL concentration of the plant extract. A similar trend was observed for the tea extract in relation to the α-glucosidase enzyme. The Vmax and Km values calculated as 0.218 ΔOD/Min and 2.266 µg/mL at 500 µg/mL decreased to 0.158 ΔOD/Min and 1.753 µg/mL at 1000 µg/mL. Overall, these results suggest uncompetitive inhibition of the hydrolytic enzymes by the chemical constituents of the tea.

**Figure 3:**
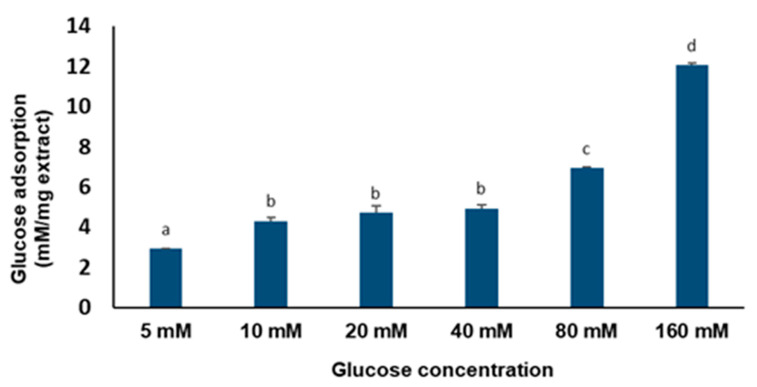
Glucose binding capacity of *C. gratissimus* tea at different concentrations of glucose. Data = mean ± SD; n = 3. ^abcd^ Values with different letters above the bars for a given concentration are significantly (*p* < 0.05) different from each other.

The glucose binding capacity of the *C. gratissimus* infusion at increasing glucose concentrations is represented in Figure 3. The tea effectively (*p* < 0.05) adsorbed glucose at different molar concentrations of glucose, ranging from 5–160 mM. There was, however, no significant (*p* > 0.05) difference in the plants’ adsorption capacity between 10 mM and 40 mM concentrations.

**Figure 4:**
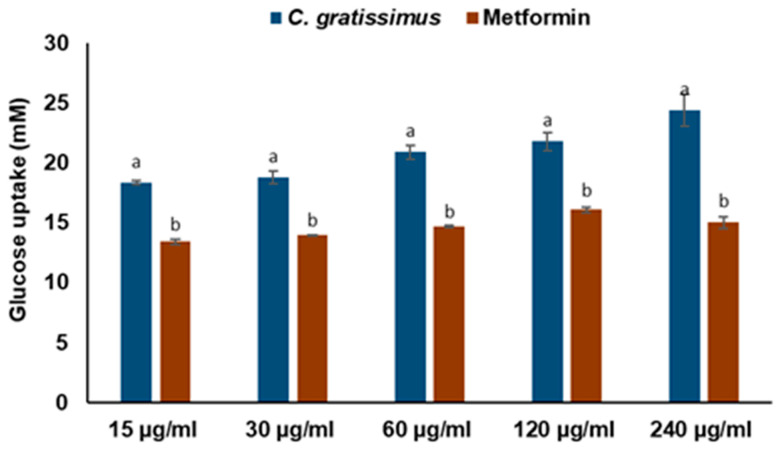
Effect of *C. gratissimus* tea on glucose uptake by yeast cells. Data = mean ± SD; n = 3. ^ab^ Values with different letters above the bars for a given concentration are significantly (*p* < 0.05) different from each other.

As depicted in Figure 4, there was a significant (*p* < 0.05) increase in the glucose uptake in yeast cells incubated with increasing concentrations of *C. gratissimus* tea, even at a better activity than metformin used as the standard drug in this assay.

**Figure 5:**
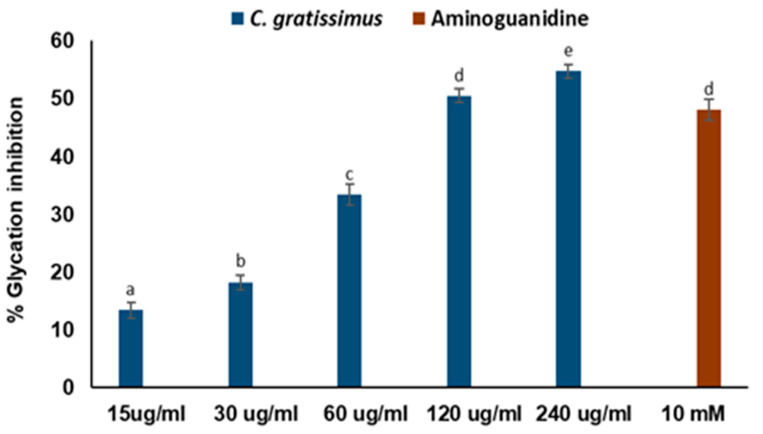
*C. gratissimus* tea inhibition of glycation as compared to a standard antiglycation drug. Data = mean ± SD; n = 3. ^abcde^ Values with different letters above the bars for a given concentration are significantly (*p* < 0.05) different from each other.

In a dose-dependent manner, *C. gratissimus* infusion significantly (*p* < 0.05) inhibited glucose-induced glycation of BSA and ensuing AGE formation, as presented in Figure 5. The anti-glycation capacity of the tea at 240 µg/mL concentration was more potent (*p* < 0.05) than that exhibited by the standard drug, 10 mM aminoguanidine.

**Figure 6:**
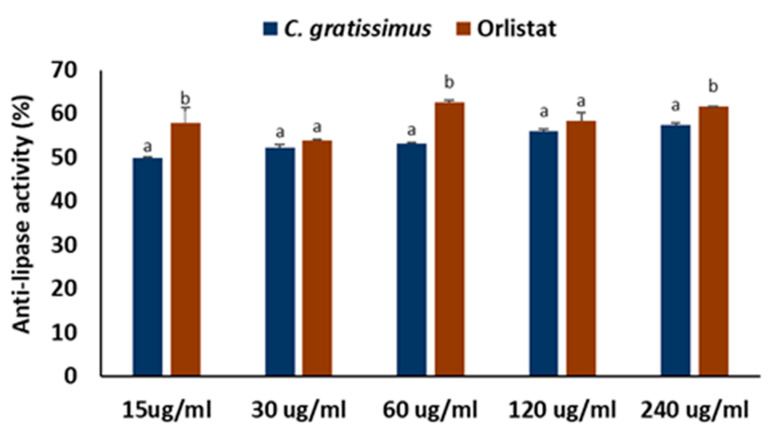
Pancreatic lipase inhibitory activities of *C. gratissimus* tea. Data = mean ± SD; n = 3. ^ab^ Values with different letters above the bars for a given concentration are significantly (*p* < 0.05) different from each other.

*C. gratissimus* tea infusion significantly (*p* < 0.05) inhibited pancreatic lipase activity as indicated in Figure 6 at an IC_50_ value of 50.27 ± 1.51 µg/mL (Table 2) and compared favorably with the standard drug, orlistat (33.05 ± 2.81 µg/mL), especially at concentrations 30 and 120 µg/mL.

**Figure 7:**
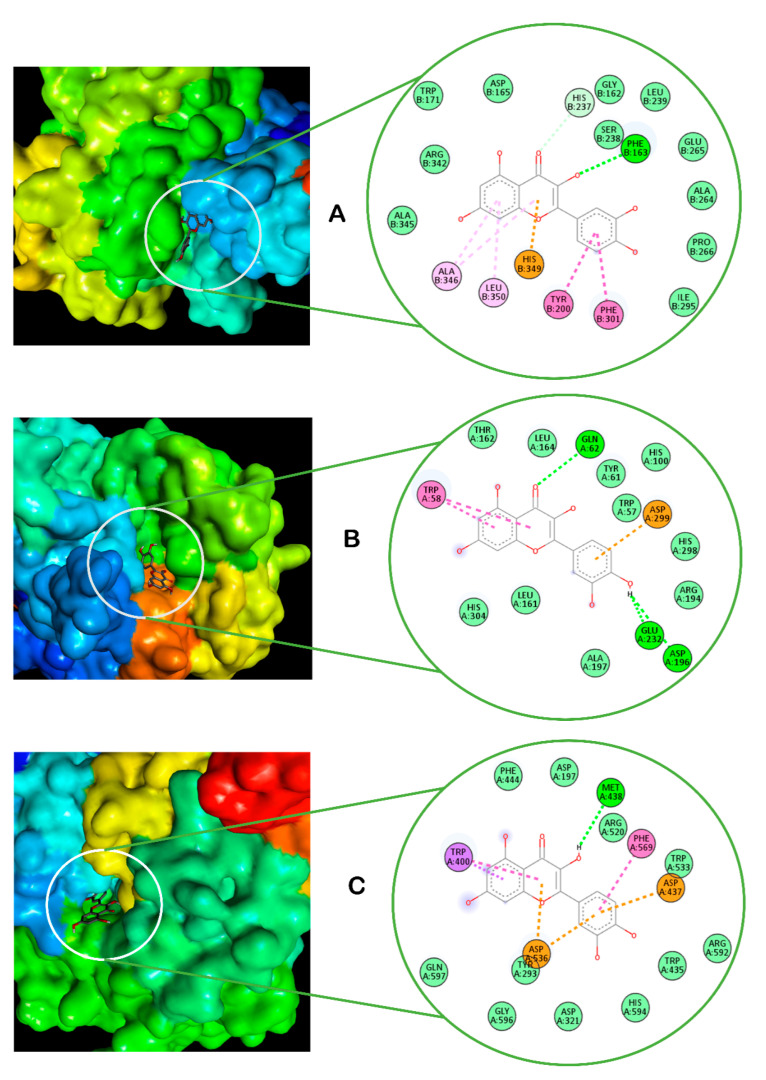
The 3D and 2D images of the molecular interactions of quercetin with the active site amino residues of (**A**) lipase, (**B**) amylase, and (**C**) glucosidase.

The molecular docking calculation of the binding energy of the tea infusion phenolic phytoconstituents and the amino residues of digestive proteins is shown in Table 4. It could be observed that quercetin (−9.7 Kcal/mol) and catechin (−9.2 Kcal/mol) had the lowest binding affinity with lipase. While quercetin similarly produced the lowest energy of attraction with α-amylase (−9.0 Kcal/mol) and α-glucosidase (−6.3 Kcal/mol), catechin had the second strongest affinity (−8.9 Kcal/mol) with the former enzyme, whereas it was caffeic acid (−6.2 Kcal/mol) for the last carbohydrate hydrolyzing enzyme. Since quercetin produced the strongest affinity for the selected digestive proteins, the 2D images of its interactions with the various enzymes are presented in Figure 7. The main force responsible for the stability of this compound in the proteins’ crystallographic structures is hydrogen bonding. This bond was formed between the compound and PHE163 of lipase, GLU232, ASP196, and GLN62 of α-amylase, as well as MET438 of α-glucosidase. Other prominent bonds that could have contributed to the compound’s molecular interactions with the various proteins include pi-anion bonds, pi-stacked bonds, and pi-sigma bonds.

**Figure 8:**
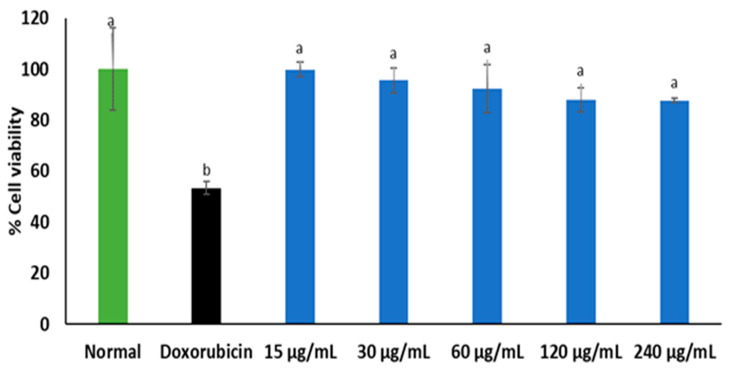
Cytotoxic effect of *C. gratissimus* on the 3T3 fibroblast cell line. Value = mean ± SD; n = 3. ^a^ Statistically significant compared to the doxorubicin group; ^b^ statistically significant compared to the normal control cell (*p* < 0.05, Tukey’s HSD-multiple range post hoc test).

The effect of *C. gratissimus* infusion on cell viability is presented in Figure 8. Though there was no significant (*p* < 0.05) difference in the cell viability of *C. gratissimus*-treated cells in comparison with the normal (untreated) 3T3 cells, there was a slight decrease in the cell viability at increasing concentrations of the extract. The cell viability of Doxorubicin-treated cells was significantly reduced as compared with normal (untreated) cells.

## 3. Discussion

The rate at which diabetes is increasing cannot be overstated, and its significant impact on global morbidity and mortality is burdensome. Over the years, traditional medicine has relied on herbal mixtures, teas, and decoctions as alternatives to managing different diseases, including diabetes [17]. Medicinal plants have attracted considerable attention due to their reservoir of therapeutic phytochemicals, safety, accessibility, and affordability. Thus, modern science targets active plant phytocompounds for the development of drugs. In the present study, the anti-hyperglycemic and anti-lipidemic activities of *C. gratissimus* herbal tea infusion, as well as the possible polyphenolic components that may be responsible for these activities, were investigated.

The therapeutic potentials of medicinal plants emanate from a wide group of bioactive compounds identified as secondary metabolites, which are classified according to their functional groups, sizes, and structures. One of such groups is the polyphenol group [16]. Studies have reported the presence of polyphenols and flavonoids in *C. gratissimus* extracts [12,18]. In agreement with these previous findings, the HPLC analysis carried out in our study (Table 1) suggests the presence of polyphenols and flavonoids in the herbal product. Interestingly, the role of these secondary metabolites in the prevention and management of diabetes and obesity has been well documented [19,20].

One of the crucial and common therapeutic targets for managing diabetes is to curb postprandial hyperglycemia by inhibiting dietary carbohydrate digestion. Pancreatic alpha-amylase hydrolyzes dietary carbohydrates into simple monosaccharides in the digestive system, while alpha-glucosidase further degrades them to glucose, which is absorbed into the blood stream. Thus, inhibition of carbohydrate digestive enzymes is a desirable mechanism for controlling postprandial hyperglycemia by preventing excess absorption of glucose, which has been used in the development of antidiabetic drugs such as acarbose [21,22]. Moreover, previous reports [23] have described the importance of understanding the mechanism of inhibition of druggable chemical compounds in enhancing their physiological target specificity as well as their efficacy profile. Although the strong binding energies and molecular interactions between the tea’s HPLC-quantified polyphenols and the proteins’ active site amino acids (Table 4 and Figure 7) may explain the basis of α-glucosidase (Figure 1A) and α-amylase (Figure 2A), the kinetic results in Figure 1B and Figure 2B suggest an uncompetitive mode of enzyme inhibition that could have been mediated by other unidentified components of the plant. Complete inhibition of carbohydrate digestive enzymes could cause polysaccharide accumulation in the small intestine, hence the development of bloating or flatulence. Therefore, the utilization of clinically approved carbohydrate digestive enzyme inhibitors like acarbose in diabetes disease management is not to totally stop polysaccharide breakdown but to slow down dietary glucose release and control postprandial glucose spike [24]. Consequently, our results corroborate previous in vivo experiments where *C. gratissimus* leaf fractions suppressed α-amylase and α-glycosidase biological activities in Wistar rats [25].

To further buttress the postprandial glucose-lowering property of *C. gratissimus* herbal tea, its glucose-binding capacity was analyzed. Medicinal plants provide an ample source of dietary supplements for improving glucose homeostasis and preventing diabetes and its long-term complications [26]. In this study, different concentrations of *C. gratissimus* were able to effectively bind with glucose (Figure 3). The glucose-binding capacity of the tea was directly proportional to the concentration of glucose. Studies have shown that one of the mechanisms by which dietary plant fibers suppress elevated blood glucose is by reducing the absorbable glucose concentration in the small intestine. Glucose adsorption is one of the in vitro screening methods of plant effectiveness for diabetes prevention [27]. The possible presence of soluble and insoluble dietary fibers in *C. gratissimus* may be responsible for its absorption properties [28]. Thus, the data suggest that *C. gratissimus* is capable of decreasing the level of glucose to be transported across the intestinal lumen, thereby suppressing postprandial hyperglycemia.

One of the well-studied in vitro glucose-lowering screenings in plants and their phytoconstituents is the mechanism of glucose transport across the yeast cell membrane [29]. It was revealed in the present study that *C. gratissimus* remarkably facilitated glucose across the yeast cell system at varying glucose concentrations (Figure 4). Glucose uptake by yeast cells is a measure of the quantity of unused glucose left in the medium after a specific time interval [30]. Studies have shown that, contrary to the mechanism of glucose uptake in eukaryotes and human cells, transport of glucose across the yeast cell (*Saccharomyces cerevisiae*) membrane is achieved by facilitated diffusion, which can only occur if there is an effective reduction (utilization) of intracellular glucose [29,31]. Therefore, the ability of *C. gratissimus* to promote glucose uptake across the yeast membrane suggests its capability of facilitating glucose utilization, thereby reducing hyperglycemia and regulating glucose homeostasis.

Incessant hyperglycemia has been implicated in the development and accumulation of stable deleterious compounds known as advanced glycation end products (AGEs) via enzymatic glycation of proteins. AGEs are highly reactive compounds that can interact with specific AGE receptors (RAGE) localized in the plasma membrane, leading to impaired intracellular signaling, proinflammatory molecule generation, altered gene expression, and oxidative stress. The accumulation of AGEs is cytotoxic and therefore implicated in a cascade of diabetic complications [5,32]. Thus, regulation of postprandial glucose is crucial for the prevention of diabetes complications [22]. In the present study, the anti-glycation effect of *C. gratissimus* tea (Figure 5) was quantified by the inhibition of AGE formation. The ability of *C. gratissimus* tea to inhibit the formation of AGE suggests its possible protective effect against the development of diabetes-induced complications. The anti-glycation activity of *C. gratissimus* tea may be attributed to its polyphenolic components (Table 3), as the role of polyphenols as glycation inhibitors has been reported [33].

Overweight and obesity are major drivers of chronic diseases such as diabetes [34]. Pancreatic lipid accumulation can impair the stimulation of pancreatic β cells’ insulin secretion as a result of lipolysis. Hence, excess free fatty acid plays a major role in insulin resistance, inhibition of glucose uptake, and suppression of glycogenolysis, which further intensifies hyperglycemia [35]. Inhibition of pancreatic lipase by inhibitors such as orlistat is a principal therapeutic mechanism for suppressing the digestion of dietary triacylglycerols (lipolysis) and a strategy for treating obesity and hyperlipidemia [36]. In the present study, the inhibition of lipase activity by the herbal tea (Figure 6) suggests the potential of *C. gratissimus* to delay lipolysis and reduce lipid absorption, thus indicating its anti-lipidemic and anti-obesogenic activity. The anti-lipidemic activity of the tea may be a result of its polyphenolic contents (Table 3), and the strong binding affinities between the phenolic acids and pancreatic lipase enzymes (Table 4 and Figure 8) may further portray the anti-lipidemic mechanism of *C. gratissimus*. Reduction of postprandial hyperglycemia retards the synthesis and accumulation of triacylglycerol [37]. The glucose-lowering effect exhibited by *C. gratissimus* (Figure 1, Figure 2, Figure 3 and Figure 4) further corroborates these results by demonstrating both its anti-diabetic and anti-obesogenic properties.

The effect of *C. gratissimus* herbal infusion was investigated on 3T3-fibroblast cells to investigate its safety. *C. gratissimus* tea showed little or no cytotoxic effect on 3T3-cell lines (Figure 8). This portrays the safety of the tea when orally consumed. However, due to the slight decrease in cell viability observed at increasing concentrations, caution must be taken at higher concentrations to avoid cytotoxicity. Thus, further studies on safety are required to ascertain the safest dose of the tea.

## 4. Materials and Methods

### 4.1. Collection of Plants and Verification

*Croton gratissimus* var. *gratissimus* (Moologa) leaves were collected from Mokgola, Northwest Province, South Africa, with written permission from the late Chief Bonnabothata Moiloa and the Mokgola Tribal Council, by Prof. M.G. Matsabisa and deposited at the Bolus Herbarium of the University of the Free State, Bloemfontein 9300, South Africa. It was identified and authenticated with a voucher specimen number (MGM009). This research is a collaborative research project with the Mokgola Community.

### 4.2. Extraction of Croton gratissimus Herbal Tea

A pack of *Croton gratissimus* (Moologa) herbal tea was obtained from the Indigenous Knowledge System’s (IKS) laboratory, Department of Pharmacology at the University of the Free State, Bloemfontein, South Africa. Twelve tea bags (20.5 g) were taken from the pack, and the contents were placed in 200 mL of boiled water and allowed to stand for 20 min while continuously stirring with a spatula. It was then sieved into a pre-weighed beaker and concentrated in a water bath (<50 °C) for hours. The beaker with the content was then weighed, which yielded an extract of 3.9 g (20.9% yield of a sticky dark brown extract) and stored in airtight glass vials until use.

A stock solution of 2 mg/mL of the infusion was made with distilled water, and working concentrations between 15 and 240 μg/mL were prepared for subsequent assays.

### 4.3. High-Performance Liquid Chromatography (HPLC) Quantification of C. gratissimus Herbal Infusion Polyphenols

The separation of polyphenols was performed with an Agilent 1100 series HPLC system equipped with an online 1260 degasser (G 1322A), a quadpump (G 1311A), an autosampler (G 1313A), a column heater (G 1316A), and a UV-Vis Diode Array Detector (DAD) (G 1315B). Instrument control and data analysis were carried out using an open lab control panel (version 3.2.2.14) through Windows 10. Separation was achieved by a Phenomenax reversed phase C18 column (5 μm particle size, 150 × 4.6 mm). The mobile phase consisted of a 1% aqueous acetic acid solution (A) and 100% methanol (B). The column temperature was maintained at 25 °C. Chromatograms were acquired at three different wavelengths (254, 280, and 300 nm) according to the absorption maxima of the analyzed compounds. Each compound was identified by its retention time and by spiking with standards under the same conditions. Samples and standards including gallic acid, catechin, caffeic acid, and quercetin were eluted according to Nour et al. [38] and Stoenescu et al. [39] with slight modifications using two different run times and conditions as follows:

First condition: 25% B from 0 to 5 min; from 25 to 30% B in 10 min; from 30 to 45% B in 16 min; 45% B from 16 to 18 min; 45 to 80% B in 25 min; 80% B for 30 min; and 80 to 25% B in 35 min to re-establish the initial conditions before the injection of another sample. The flow rate was 0.5 mL/min, and the injection volume was 10 μL.

Second condition: 90% A and 10% B from 0 to 6 min; 84% A and 16% B from 7 to 25 min; 72% A and 28% B from 26 to 37 min; 65% A and 35% B from 38 to 47 min; 50% A and 50% B from 48 to 64 min; and 90% A and 10% B from 65 to 70 min, to restore the initial conditions, before injection of a new sample. The flow rate was 0.8 mL/min, and the injection volume was 5 μL.

### 4.4. In Vitro Enzyme Inhibition

#### 4.4.1. α-Glucosidase Inhibition

The inhibitory activity of the *C. gratissimus* infusion was determined according to Ademiluyi and Oboh [40], with slight modifications. In brief, 100 µL of varying concentrations of the infusion (15–240 μg/mL) or acarbose was incubated with 100 µL of 1 U/mg α-glucosidase solution for 15 min at 37 °C. Moreover, 40 µL of the substrate, pNPG (5 mM) in phosphate buffer (PH 6.8; 100 mM) was then added and incubated for an additional 30 min. The absorbance of the generated p-nitrophenol was quantified at 405 nm using a Multiskan Go, UV spectrophotometer (Thermo Scientific, Ratastie, Finland). The assay was carried out in triplicate. The percentage inhibition of α-glucosidase was expressed as follows:

$$\%Inhibition=\frac{\left(Absorbance\;of\;control-Absorbance\;of\;test\;Sample\right)}{\left(Absorbance\;of\;control\right)}\times 100$$

#### 4.4.2. α-Amylase Inhibition

The inhibitory activity of the *C. gratissimus* infusion was determined according to Ademiluyi and Oboh [40], with slight modifications. Briefly, 300 μL of the extract or acarbose (15–240 μg/mL) was incubated with 300 μL of 4 U/mL pancreatic amylase in 100 mM sodium phosphate buffer (pH 6.8) for 15 min at 37 °C. Moreover, 200 μL of 1% starch dissolved in the assay buffer was then added to the reaction mixture and incubated further for 30 min at 37 °C. After incubation, 100 mL of dinitrosalicylate (DNS) color reagent was added to the mixture and boiled for 10 min. Absorbance was measured at 540 nm, and the α-amylase inhibitory activity of *C. gratissimus* was expressed as a percentage of control without the inhibitors.

#### 4.4.3. Porcine Pancreatic Lipase Inhibition

A modified method of Kim et al. [41] was used to analyze the inhibitory effect of *C. gratissimus* on pancreatic lipase. Briefly, 2.5 mg/mL porcine pancreatic lipase dissolved in a buffer system (3-(N-morpholino) propane sulfonic acid and EDTA at pH 6.8) was centrifuged for 20 min at 15,000× *g*. Moreover, 100 μL of the enzyme supernatant was then incubated with 500 μL of the extract or Orlistat (standard inhibitor) and 350 μL of Tris buffer (100 mM Tris-HCl and 5 mM CaCl_2_, pH 7.0) for 15 min at 37 °C. The experimental control lacked the inhibitor (extract). After incubation, 50 μL of the substrate, p-nitrophenyl butyrate (10 mM), was added and further incubated for 10 min. Lipase inhibition was determined by quantifying the hydrolysis of p-nitrophenyl butyrate to p-nitrophenol at 405 nm. Lipase inhibitory activity was expressed as a percentage of the experimental control lacking inhibitors/infusions.

### 4.5. Inhibition Kinetics of Carbohydrate Digestive Enzymes

#### 4.5.1. Mode of α-Glucosidase Inhibition

The mode of α-glucosidase inhibition by *C. gratissimus* herbal tea was evaluated using a modified method of Kazeem et al. [42] based on increasing substrate concentration and a constant concentration of enzyme with or without the inhibitor (extract). Briefly, in a set of 6 tubes, 50 μL of 500 or 1000 μg/mL concentration of *C. gratissimus* were pre-incubated with 100 μL of α-glucosidase (1.0 U/mL) for 10 min at 37 °C. In another tube set, 50 μL of phosphate buffer (100 mM; pH 6.9) was pre-incubated with α-glucosidase solution. Moreover, 30 μL of the substrate, p-Nitrophenyl-α-D-glucopyranoside (0.156–5.0 mM), was added to the sets of solution to initiate the reaction, which was incubated for 5 min at 37 °C. The change in absorbance of the generated p-nitrophenol was monitored for 4 min at 405 nm using a Multiskan Go, UV spectrophotometer (Thermo Scientific, Ratastie, Finland). The type of inhibition was determined by the analysis of a Lineweaver–Burk double reciprocal plot constructed from the reaction velocities (V_o_) and concentrations of substrates (S). The Michaelis–Menten constant (Km) and maximum rate of enzyme reactions (V_max_) were calculated from the plots according to the expression below.

1/V_o_ = Km/V_max_[S] + 1/V_max_.

#### 4.5.2. Mode of α-Amylase Inhibition

α-Amylase inhibition kinetics by *C. gratissimus* herbal tea were determined according to a previously established method [42], with modifications. Briefly, 200 μL of 500 or 1000 μg/mL concentration of *C. gratissimus* were pre-incubated with 150 μL of 4.0 U/mL α-amylase solution for 10 min at 37 °C. In another set, 200 μL of 100 mM phosphate buffer (pH 6.9) was pre-incubated with the enzyme solution. After incubation, 150 μL of starch solution (0.063–0.5%) was included in all sets of the reaction mixture and incubated for 10 min at 37 °C. Moreover, 200 μL of dinitrosalicylic acid (DNSA) was used to terminate the reaction and boiled for 10 min. The change in absorbance of the amount of released reducing sugar was monitored spectrophotometrically for 5 min at 540 nm. The inhibition mode of α-amylase inhibition by *C. gratissimus* was determined as explained above.

### 4.6. Advanced Glycation End-Products (AGEs) Inhibition

The inhibition of protein glycation assay was carried out according to a modified method of Grzegorczyk-Karolak et al. [43]. In brief, 500 μL of 1 mg/mL bovine serum albumin (BSA) was incubated with 100 μL equal volumes of 0.5 M glucose and 0.5 M fructose in phosphate-buffered saline (pH 7.4) and 100 μL of the extracts or aminoguanidine (a standard glycation inhibitor) in the dark for 4 days at 60 °C. The experimental control has no inhibitor (extract). AGEs formation was estimated using fluorescent properties at excitation and emission wavelengths of 370 nm and 440 nm, respectively. Percentage glycation inhibition was expressed as follows:

$$\%AGE\;Inhibition=\frac{\left(Fluorescence\;of\;control-Fluorescence\;of\;test\;Sample\right)}{\left(Fluorescence\;of\;control\right)}\times 100$$

### 4.7. Glucose Adsorption Capacity of C. gratissimus

The glucose adsorption capacity of *C. gratissimus* was determined in vitro according to the method of Bhinge et al. [30], with a slight modification. Briefly, 1% of the herbal tea extract was added to various concentrations (0, 5, 10, 20, 40, 80, and 100 mM) of 25 mL glucose and incubated in a water bath with a shaker for 6 h (37 °C). After incubation, the mixtures were centrifuged for 20 min (4000× *g*), and the bound glucose concentration was quantified using the dinitrosalicylic acid method and calculated using the expression below.

$$Glucose\;bound=\frac{\left(GB-GA\right)}{\left(Weight\;of\;sample\right)}\times Volume\;of\;solution$$

where GB is the glucose concentration before incubation and GA is the glucose concentration after 6 h of incubation.

### 4.8. Cell Lines and Cytotoxicity Screening

#### 4.8.1. Cell Maintenance

NIH-3T3 fibroblast cell lines were obtained from Cellonex, South Africa. Cells were maintained in DMEM supplemented with 10% FBS (Gibco, Thermofisher, Johannesburg, South Africa) and incubated at 37 °C in a humidified environment with 5% CO_2_. The cells were subcultured every other day.

#### 4.8.2. Cytotoxicity Screening

After dissociating the cells with trypsin, the cells were counted with the Countess II Automated Cell Counter (Thermofisher, Johannesburg, South Africa) and then seeded at a density of 1 × 10^4^ cells per well (and 100 µL of media per well) in a 96-well plate. Cells were allowed to attach overnight at 37 °C with 5% CO_2_. The media was discarded, and the cells were treated with 15–240 µg/mL concentrations of *C. gratissimus* for 48 h at 37 °C with 5% CO_2_. Thereafter, the spent media was discarded and replaced with 100 µL of MTT solution (100 µL per well) and further incubated for 2 h at the initial condition. The same volume of 100% DMSO was used to replace the MTT solution. Subsequently, the absorbance was recorded at 550 nm using the Multiskan GO spectrophotometer (Thermo Scientific, Ratastie, Finland). Doxorubicin (3 µg/mL; Sigma Aldrich, Johannesburg, South Africa) served as the positive control.

### 4.9. Glucose Transport/Uptake by Yeast Cells

A previously established method by Nirupama et al. [44] was used to evaluate the effect of *C. gratissimus* tea on glucose transport across yeast cells, with a slight modification. This was evaluated by quantifying the decrease in the amount of glucose in a reaction mixture of yeast cell suspension and extract. Briefly, a total volume of 500 μL of each varying concentration (15–240 μg/mL) of the extract in distilled water was incubated with 500 μL of 50 mM glucose solution for 10 min at 37 °C. Thereafter, an aliquot of 100 μL of yeast suspension (1%) was added to the mixture and further incubated at the same conditions for 1 h. The final amount of glucose in the mixtures was determined using the dinitrosalicylic acid method, and the results were extrapolated from a glucose standard (0–50 mM) curve.

### 4.10. Molecular Docking

Compounds quantified from the *C. gratissimus* aqueous infusion via HPLC analysis were subjected to molecular docking simulation using the Autodock Vina algorithm of UCFS Chimera software V. 1.14. The 3D crystallographic structure of the α-amylase (1B2Y) complexed acarbose standard drug, α-glucosidase (3CTT) bonded with casuarina, and lipase (Pancreatic) with access code 1LPB were retrieved in PDB format from the protein data bank, accessible at https://www.rcsb.org/ (accessed on 26 April 2024). Then, the protein refinement procedure carried out with the Dockprep tool of the same software involves removing the water atoms co-crystallized with the structure of other non-protein molecules. After further adding Gasteiger charges, the resulting minimized refined protein with the lowest stable conformation was saved in mol2 file format for docking analysis with the plant-infusion annotated chemical compounds obtained from the PubChem NCBI database. Once the latter ligands (compounds) were prepared via a similar procedure employed for the protein molecules, they were docked at the active site of the protein in a search grid with X, Y, and Z dimensions of 18.9 × 5.80 × 47 for α-amylase, 1.33 × −15.72 × 20.25 for α-glucosidase, and 10 × 22 × 51 for lipase. While the active site location of the proteins was determined with the CASTp online server (http://sts.bioe.uic.edu/castp/calculation.html accessed on 26 April 2024), the 2D and 3D images of the ligand–protein complex with the lowest binding energy for compounds and the protein were inspected with BIOVIA Discovery Studio software (V21.1.0.20298).

### 4.11. Statistical Analysis

Statistical analyses were performed using SPSS (Windows V25). Data were expressed as mean ± SD with experiments carried out in triplicate (n = 3). Statistically significant differences between the groups were considered at *p* < 0.05.

## 5. Conclusions

Data from the present study demonstrate the in vitro mechanisms by which *C. gratissimus* herbal tea regulates glucose homeostasis by inhibiting carbohydrate and fat digestive enzymes, retarding the glycation process, facilitating glucose uptake in yeast cells, and exhibiting considerable glucose binding capacity. The results were further validated with molecular docking studies to show the effect of the polyphenol constituents of the tea on its biological activities. These results may support the local usage of *C. gratissimus* herbal tea as a potential anti-hyperglycemic and anti-obesogenic tea. Further in vivo, molecular, as well as safety and toxicology studies are encouraged to ascertain specific doses and ratify the present in vitro data.

## Figures and Tables

**Table 1:** HPLC-quantified polyphenols in *C. gratissimus* herbal tea.

Polyphenols	Parameters					
	Retention Time (min)			RT (min)	λ Max	% Peak Area
	Standard	Sample	DFS			
Gallic acid	4.753	4.691	+0.062	35	254	13.5152
Catechin	4.548	4.567	−0.019	35	254	15.6037
Caffeic acid	3.395	3.403	−0.008	70	254	16.3901
Quercetin	3.594	3.522	+0.072	70	300	15.8977

Note: RT: peak retention time; λ max: scanning wavelength; DFS: the difference between the peak retention time of the standard polyphenol and the peak retention time of that shown in the HPLC chromatogram of *Croton gratissimus* tea leaf extract.

**Table 2:** IC_50_ values of *C. gratissimus* herbal tea digestive enzyme inhibitory activities.

Activities	*C. gratissimus*	Acarbose	Orlistat
α-Glucosidase	60.56 ± 2.78	25.21 ± 1.19	-
α-Amylase	35.67 ± 0.07	9.53 ± 0.18	-
Pancreatic lipase	50.27 ± 1.51	-	33.05 ± 2.81

All values are expressed in µg/mL.

**Table 3:** Inhibition kinetics of the *C. gratissimus* tea infusion on carbohydrate digestive enzymes.

	α-Amylase		α-Glucosidase	
	Vmax (ΔOD/Min)	Km (µg/mL)	Vmax (ΔOD/Min)	Km (µg/mL)
0 µg/mL Infusion	0.079	6.311	0.426	12.06
500 µg/mL Infusion	0.087	19.075	0.218	2.266
1000 µg/mL Infusion	0.046	2.614	0.158	1.753

**Table 4:** Binding energies (Kcal/mol) of HPLC-quantified polyphenolic compounds of *C. gratissimus* tea.

Polyphenols	Alpha-Glucosidase	Alpha-Amylase	Lipase
Caffeic acid	−6.2	−6.5	−6.7
Gallic acid	−6.0	−6.2	−6.0
Catechin	−5.4	−8.9	−9.2
Quercetin	−6.3	−9.0	−9.7

All values are expressed in Kcal/mol.

## Data Availability

Data are contained within the article.

## References

[1] Dilworth L., Facey A., Omoruyi F. (2021). Diabetes mellitus and its metabolic complications: The role of adipose tissues. Int. J. Mol. Sci..

[2] IDF (2021). International Diabetes Federation (IDF) Diabetes Atlas.

[3] Cheng R., Ma J.-X. (2015). Angiogenesis in diabetes and obesity. Rev. Endocr. Metab. Disord..

[4] Leitner D.R., Frühbeck G., Yumuk V., Schindler K., Micic D., Woodward E., Toplak H. (2017). Obesity and type 2 diabetes: Two diseases with a need for combined treatment strategies-EASO can lead the way. Obes. Facts.

[5] Beseni B.K., Bagla V.P., Njanje I., Matsebatlela T.M., Mampuru L., Mokgotho M.P. (2017). Antioxidant, antiglycation, and hypoglycaemic effect of seriphium plumosum crude plant extracts. Evid. Based Complement. Altern. Med..

[6] Oyedemi S., Koekemoer T., Bradley G., van de Venter M., Afolayan A. (2013). In vitro anti-hyperglycemia properties of the aqueous stem bark extract from *Strychnos henningsii* (Gilg). Int. J. Diabetes Dev. Ctries..

[7] Moremi M.P., Kamatou G.P., Viljoen A.M., Tankeu S.Y. (2021). *Croton gratissimus*-essential oil composition and chemometric analysis of an ethnomedicinally important tree from South Africa. S. Afr. J. Bot..

[8] Okonkon J., Bassey A., Obot J. (2006). Antidiabetic activity of ethanolic leaf extract of *Croton zambesicus* Muell.(thunder plant) in alloxan diabetic rats. Afr. J. Trad. Complement. Altern. Med..

[9] Erhabor J.O., Oyenihi O.R., Erukainure O.L., Matsabisa M.G. (2022). Croton gratissimus Burch.(*Lavender croton*): A Review of the Traditional Uses, Phytochemistry, Nutritional Constituents and Pharmacological Activities. Trop. J. Nat. Prod. Res..

[10] Mulholland D.A., Langat M.K., Crouch N.R., Coley H.M., Mutambi E.M., Nuzillard J.-M. (2010). Cembranolides from the stem bark of the southern African medicinal plant, *Croton gratissimus* (Euphorbiaceae). Phytochemistry.

[11] Mthethwa N.S., Oyedeji B.A., Obi L.C., Aiyegoro O.A. (2014). Anti-staphylococcal, anti-HIV and cytotoxicity studies of four South African medicinal plants and isolation of bioactive compounds from *Cassine transvaalensis* (Burtt. Davy) codd. BMC Complement. Altern. Med..

[12] Ndhlala A.R., Aderogba M.A., Ncube B., Van Staden J. (2013). Anti-oxidative and cholinesterase inhibitory effects of leaf extracts and their isolated compounds from two closely related Croton species. Molecules.

[13] Ncume P.V., Salau V.F., Mtshali S., Olofinsan K.A., Erukainure O.L., Matsabisa M.G. (2023). Phytochemical Properties of *Croton gratissimus* Burch (Lavender Croton) Herbal Tea and Its Protective Effect against Iron-Induced Oxidative Hepatic Injury. Plants.

[14] Ofusori D.A., Komolafe O.A., Adewole O.S., Obuotor E.M., Fakunle J.B., Ayoka A.O. (2012). Effect of ethanolic leaf extract of *Croton zambesicus* (Müll. Arg.) on lipid profile in streptozotocin-induced diabetic rats. Diabetol. Croat..

[15] Magwilu K.D., Nguta J.M., Mapenay I., Matara D. (2022). Phylogeny, phytomedicines, phytochemistry, pharmacological properties, and toxicity of *Croton gratissimus* Burch (Euphorbiaceae). Adv.Pharm. Pharm. Sci..

[16] Mohamed A.I., Beseni B.K., Msomi N.Z., Salau V.F., Erukainure O.L., Aljoundi A., Islam M.S. (2022). The antioxidant and antidiabetic potentials of polyphenolic-rich extracts of *Cyperus rotundus* (Linn.). J. Biomol. Struct. Dyn..

[17] Matsabisa M., Chukwuma C., Chaudhary S. (2019). South African traditional herbal formulation inhibits α-glucosidase, DPP-IV and glycation activities, and modulates glucose utilisation in Chang liver cells and 3T3-L1 adipocytes. S. Afr. J.Bot..

[18] Mahmoud A.B., Danton O., Kaiser M., Khalid S., Hamburger M., Mäser P. (2020). HPLC-based activity profiling for antiprotozoal compounds in *Croton gratissimus* and *Cuscuta hyalina*. Front. Pharmacol..

[19] Salau V.F., Erukainure O.L., Islam M.S. (2020). Phenolics: Therapeutic applications against oxidative injury in obesity and type 2 diabetes pathology. Pathology.

[20] Wang Y., Alkhalidy H., Liu D. (2021). The emerging role of polyphenols in the management of type 2 diabetes. Molecules.

[21] Alqahtani A.S., Hidayathulla S., Rehman M.T., ElGamal A.A., Al-Massarani S., Razmovski-Naumovski V., Alqahtani M.S., El Dib R.A., AlAjmi M.F. (2019). Alpha-amylase and alpha-glucosidase enzyme inhibition and antioxidant potential of 3-oxolupenal and katononic acid isolated from *Nuxia oppositifolia*. Biomolecules.

[22] Ghadyale V., Takalikar S., Haldavnekar V., Arvindekar A. (2012). Effective control of postprandial glucose level through inhibition of intestinal alpha glucosidase by *Cymbopogon martinii* (Roxb.). Evid-Based Complement. Altern. Med..

[23] Cele N., Awolade P., Seboletswe P., Olofinsan K., Islam M.S., Singh P. (2022). α-Glucosidase and α-amylase inhibitory potentials of quinoline–1, 3, 4-oxadiazole conjugates bearing 1, 2, 3-triazole with antioxidant activity, kinetic studies, and computational validation. Pharmaceuticals.

[24] Creutzfeldt W. (1999). Effects of the α-glucosidase inhibitor acarbose on the development of long-term complications in diabetic animals: Pathophysiological and therapeutic implications. Diabetes Metab. Res. Rev..

[25] Okokon J.E., Bassey A.I., Udom G.J., Edem U.A., Attah G. (2022). Alpha Amylase and Alpha Glucosidase Inhibitory Activities of *Croton zambesicus* Leaf Fractions in Wistar Rats. J. Curr. Biomed. Res..

[26] Bhutkar M., Bhinge S., Randive D., Wadkar G., Todkar S. (2018). Studies on glucose adsorption capacity of some indigenous plants. Glob. J. Pharm. Pharm. Sci..

[27] Ninomiya K., Ina S., Nakamura H., Yamaguchi Y., Kumagai H., Kumagai H. (2022). Evaluation of the amount of glucose adsorbed on water-soluble dietary fibres by the analysis of its diffusion rate through a dialysis membrane. Food Hydrocoll..

[28] Das M., Devi G. (2015). In vitro glucose binding activity of *Terminalia bellirica*. Asian J. Pharm. Clin. Res..

[29] Pitchaipillai R., Ponniah T. (2016). In vitro antidiabetic activity of ethanolic leaf extract of bruguiera *Cylindrica* L.–glucose uptake by yeast cells method. Int. Biol. Biomed. J..

[30] Bhinge S.D., Bhutkar M.A., Randive D.S., Wadkar G.H., Hasabe T.S. (2017). In vitro hypoglycemic effects of unripe and ripe fruits of Musa sapientum. Braz. J. Pharm. Sci..

[31] Rehman G., Hamayun M., Iqbal A., Ul Islam S., Arshad S., Zaman K., Ahmad A., Shehzad A., Hussain A., Lee I. (2018). In vitro antidiabetic effects and antioxidant potential of *Cassia nemophila* pods. BioMed Res. Int..

[32] Mohd Dom N.S., Yahaya N., Adam Z., Hamid M. (2020). Antiglycation and antioxidant properties of *Ficus deltoidea* varieties. Evid-Based Complemt. Alter, Med..

[33] Yeh W.-J., Hsia S.-M., Lee W.-H., Wu C.-H. (2017). Polyphenols with antiglycation activity and mechanisms of action: A review of recent findings. J. Food Drug Anal..

[34] Hou X.-D., Qin X.-Y., Hou J., Tang H., Ge G.-B. (2022). The potential of natural sources for pancreatic lipase inhibitors: A solution of the obesity crisis?. Expert Opin. Drug Discov..

[35] Salau V.F., Erukainure O.L., Koorbanally N.A., Islam M.S. (2022). Ferulic acid promotes muscle glucose uptake and modulate dysregulated redox balance and metabolic pathways in ferric-induced pancreatic oxidative injury. J. Food Biochem..

[36] Kim D.H., Park Y.H., Lee J.S., Jeong H.I., Lee K.W., Kang T.H. (2020). Anti-obesity effect of DKB-117 through the inhibition of pancreatic lipase and α-amylase activity. Nutrients.

[37] Chadt A., Al-Hasani H. (2020). Glucose transporters in adipose tissue, liver, and skeletal muscle in metabolic health and disease. Pflüg. Arch. Eur. J. Physiol..

[38] Nour V., Trandafir I., Cosmulescu S. (2013). HPLC determination of phenolic acids, flavonoids and juglone in walnut leaves. J. Chromatogr. Sci..

[39] Stoenescu A.-M., Trandafir I., Cosmulescu S. (2022). Determination of phenolic compounds using HPLC-UV method in wild fruit species. Horticulturae.

[40] Ademiluyi A.O., Oboh G. (2013). Soybean phenolic-rich extracts inhibit key-enzymes linked to type 2 diabetes (α-amylase and α-glucosidase) and hypertension (angiotensin I converting enzyme) in vitro. Exp. Toxicol. Pathol..

[41] Kim Y.S., Lee Y.M., Kim H., Kim J., Jang D.S., Kim J.H., Kim J.S. (2010). Anti-obesity effect of *Morus bombycis* root extract: Anti-lipase activity and lipolytic effect. J. Ethnopharmacol..

[42] Kazeem M., Adamson J., Ogunwande I. (2013). Modes of inhibition of α-amylase and α-glucosidase by aqueous extract of *Morinda lucida* Benth leaf. BioMed Res. Int..

[43] Grzegorczyk-Karolak I., Gołąb K., Gburek J., Wysokińska H., Matkowski A. (2016). Inhibition of advanced glycation end-product formation and antioxidant activity by extracts and polyphenols from *Scutellaria alpina* L. and *S. altissima* L. Molecules.

[44] Nirupama R., Devaki M., Nirupama M., Yajurvedi H. (2014). In vitro and in vivo studies on the hypoglycaemic potential of Ashwagandha (*Withania somnifera*) root. Pharm. Sci. Monit..

